# Statistical and machine learning methods for analysis of multiplex protein data from a novel proximity extension assay in patients with ST-elevation myocardial infarction

**DOI:** 10.1038/s41598-021-93162-3

**Published:** 2021-07-02

**Authors:** Emil Maag, Archana Kulasingam, Erik Lerkevang Grove, Kamilla Sofie Pedersen, Steen Dalby Kristensen, Anne-Mette Hvas

**Affiliations:** 1 BioXpedia A/S, Palle Juul-Jensens, Blvd 82, 8200 Aarhus N, Denmark; 2 Bioinformatics Research Centre (BiRC), Aarhus University, C.F. Møllers Allé 8, Building 110, 8000 Aarhus C, Denmark; 3 Department of Cardiology, Aarhus University Hospital, Palle Juul-Jensens Blvd 99, 8200 Aarhus N, Denmark; 4 Department of Clinical Medicine, Faculty of Health, Aarhus University, Palle Juul-Jensens Blvd 82, 8200 Aarhus N, Denmark; 5 Thrombosis and Haemostasis Research Unit, Department of Clinical Biochemistry, Aarhus University Hospital, Palle Juul-Jensens Blvd 99, 8200 Aarhus N, Denmark

**Keywords:** Myocardial infarction, Applied mathematics, Biomarkers

## Abstract

Using data from patients with ST-elevation myocardial infarction (STEMI), we explored how machine learning methods can be used for analysing multiplex protein data obtained from proximity extension assays. Blood samples were obtained from 48 STEMI-patients at admission and after three months. A subset of patients also had blood samples obtained at four and 12 h after admission. Multiplex protein data were obtained using a proximity extension assay. A random forest model was used to assess the predictive power and importance of biomarkers to distinguish between the acute and the stable phase. The similarity of response profiles was investigated using K-means clustering. Out of 92 proteins, 26 proteins were found to significantly distinguish the acute and the stable phase following STEMI. The five proteins tissue factor pathway inhibitor, azurocidin, spondin-1, myeloperoxidase and myoglobin were found to be highly important for differentiating between the acute and the stable phase. Four of these proteins shared response profiles over the four time-points. Machine learning methods can be used to identify and assess novel predictive biomarkers as showcased in the present study population of patients with STEMI.

## Introduction

The recent development of novel technologies to achieve high dimensional multiplex protein data, such as proximity extension assays, has made it possible to obtain large-scale protein biomarker data sets for a wide array of diseases and pathologies. The advent of large-scale complex data has, however, made it difficult to use conventional approaches for data analysis; thus, the use of advanced statistical and machine learning methods is highly relevant to obtain in-depth scientific knowledge and discover novel biomarkers.

In general, data analyses can be described by two different approaches. The first approach uses qualitative and more conventional methods by reviewing published literature to investigate if a specific protein has previously been identified as a candidate biomarker or associated with a specific disease or pathological state. However, this approach quickly becomes infeasible if the investigated data set is large and complex. The second approach employs quantitative methods for data analyses, such as statistical and machine learning methods like random forest^1^ or Least Absolute Shrinkage and Selection Operator (LASSO) regression^2^. Random forest and LASSO regression are predictive models used to assess the importance of a larger number of variables, such as for example protein abundance levels. Other quantitative approaches include k-means^3^ or principal component analysis^4^. K-means and principal component analysis are unsupervised statistical analyses used to group complex data through patterns that reduce the available information into a distilled format. However, quantitative methods cannot always stand alone when it comes to interpreting results; therefore, a relevant balance between the quantitative and qualitative methods is essential.

The data presented here consist of abundance levels of 92 proteins in plasma from a cohort of patients diagnosed with ST-elevation myocardial infarction (STEMI) and acutely admitted for primary percutaneous coronary intervention (PPCI). Blood samples were collected in the acute phase of STEMI and in the stable phase three months after PPCI^5^. Additional samples were collected for a subgroup four hours and 12 h after PPCI, respectively^6^. Thus, the data analysed in this study using advanced statistics consist of data previously analysed by more conventional methods^1^ supplemented with new data from two time-points between the acute and stable phase of STEMI. Kulasingam et al. presented a qualitative analysis of the data focusing on identification of significant differential expression of the multiplex protein data between the acute and the stable phase.

In the present study, we employed a random forest model^1^ to investigate the hypothesis that a minor fraction of the proteins can be used to predict whether a randomly selected sample is from the acute phase or the stable phase of STEMI. Whether a sample belongs to the acute phase or the stable phase is determined by a predefined period of time. This means that predicting whether a sample belongs to the acute phase or the stable phase does not have any direct diagnostic potential. However, as the time factor is known and the acute phase and the stable phase might be well separated, this data set presents an opportunity to showcase an approach of using statistical learning models for data generated by proximity extension assays. Furthermore, using the k-means algorithm^3^ it will be investigated if any of the measured proteins share similar response profiles.

## Methods and materials

### Patient cohort and design

This study investigated the abundance levels of 92 proteins associated with cardiovascular diseases in plasma from a cohort of 48 patients diagnosed with STEMI and acutely admitted for PPCI at Aarhus University Hospital, Denmark from October 2009 through April 2010^5,6^. Baseline characteristics of the study population are shown in Table 1. Patients were included if above 18 years of age and diagnosed with STEMI. Patients were excluded if treated with dipyridamole, non-steroidal anti-inflammatory drugs, anticoagulants or lack of giving informed consent^6^.

**Table 1:** Baseline characteristics of the study population.

	Total population (n = 48)	Patients receiving bivalirudin during PPCI(n = 16)
Age, years, mean ± standard deviation	60 ± 12	66 ± 9
Male, gender, n (%)	38 (79)	11 (69)
Smokers, n (%)	31 (65)	10 (63)
Hypertension, n (%)	23 (48)	11 (69)
Body Mass Index, kg/m2, median (interquartile range)	27 (25–30)	25 (23–27)
Diabetes, n (%)	5 (10)	3 (19)
Total plasma cholesterol, mmol/l, median (interquartile range)	5.0 (4.3–5.6)	4.9 (4.4–6.3)
Previous myocardial infarction, n (%)	6 (13)	1 (6)
Previous stroke or TIA, n (%)	1 (2)	1 (6)

More details have been provided by Funck-Jensen et al.^6^.

PPCI = primary percutaneous coronary intervention; TIA = Transient ischaemic attack.

**Figure 1:**
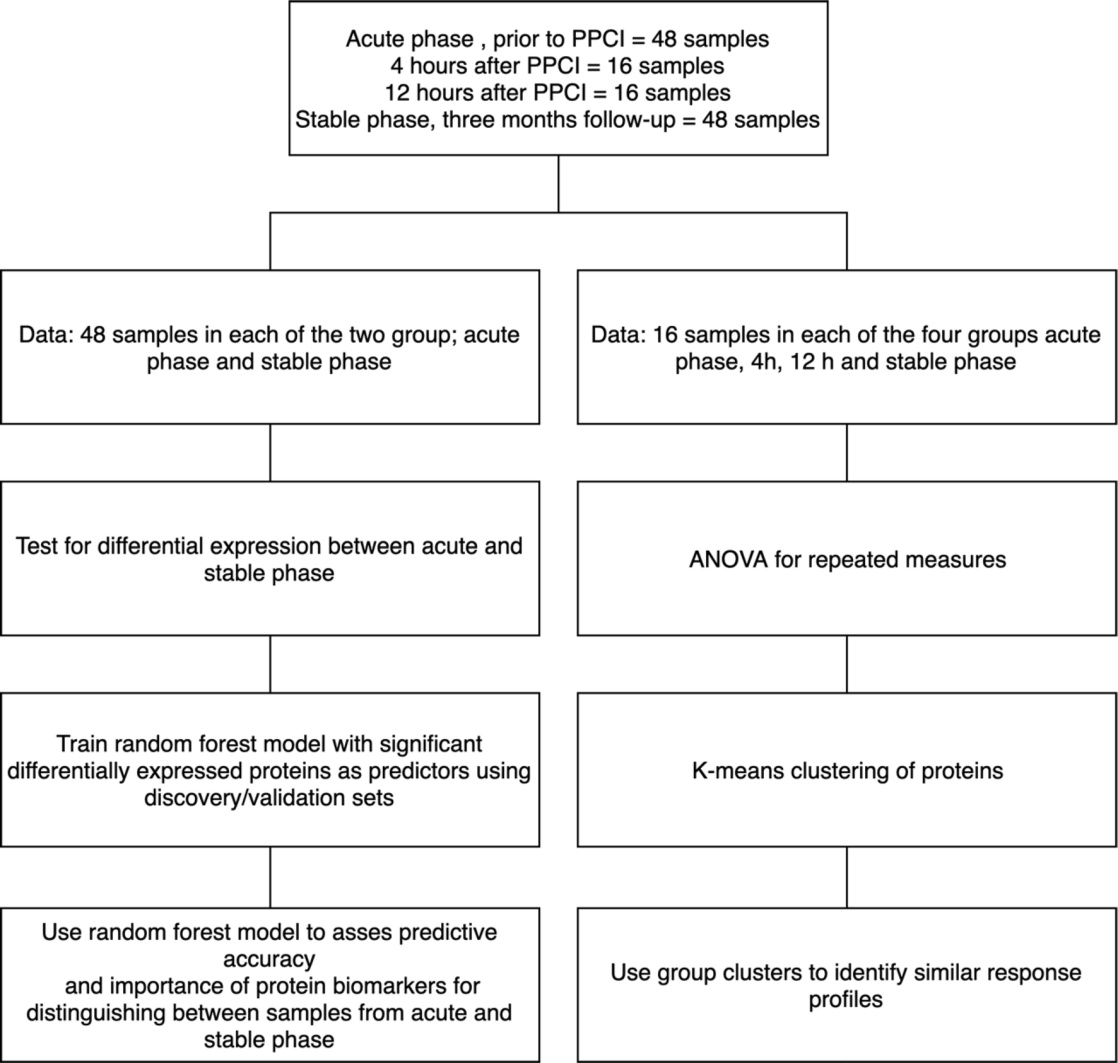
Flowchart of the statistical analysis used with the STEMI data set. The left side of the flowchart shows the flow for identifying differentially expressed proteins and assessing predictive accuracy and importance of significant proteins. The right side of the flowchart shows the flow for investigating response profiles of biomarkers from the acute to the stable phase. The figure was made in Microsoft Powerpoint version 2016.

The study was conducted according to the principles of the Declaration of Helsinki and was registered on www.clinicaltrials.gov (NCT23374). The study was approved by the Central Denmark Region Committees on Biomedical Research Ethics and the Danish Data Protection Agency. All patients gave informed consent. In brief, samples were obtained from the femoral arterial sheath prior to acute angiography at admission. For all 48 patients, blood samples were collected in the acute phase of STEMI (prior to PPCI) and in the stable phase of STEMI (three months after PPCI). In 16 of the 48 patients who received bivalirudin during PPCI, two extra blood samples were collected four and 12 h after PPCI, respectively. These samples as well as the samples collected at the three-month follow up were obtained from a cubital vein. Samples were stored at − 80 °C at Department of Clinical Biochemistry, Aarhus University Hospital, Denmark until analysis (Fig. 1).

Patients were all treated with aspirin 300 mg and clopidogrel 600 mg orally, and unfractionated heparin 10,000 IU intravenously before transferal to the catheterization laboratory for PPCI. All patients were treated with 75 mg aspirin and 75 mg clopidogrel daily as well as statins until follow-up. Also, several patients were prescribed beta blockers, angiotensin-converting-enzyme inhibitors, proton pump inhibitors or medication for diabetes^6^.

### Proteomics

The biomarker panel CARDIOVASCULAR III (Olink® Bioscience, Uppsala, Sweden) based on the proximity extension assay (PEA) technique from Olink® was used to analyse plasma samples for 92 cardiovascular disease-related proteins as listed by the manufacturer^7^. Bioanalytical analyses were performed at BioXpedia A/S, Aarhus, Denmark. The PEA technology is an immuno-PCR method enabling large-scale multiplex screening of biomarkers in targeted panels consisting of proteins in 92-plex. In brief, the method employs a specific pair of oligonucleotide-labelled antibodies that binds to each of the 92 target proteins. Dual binding in proximity of matching antibody-probes results in hybridization of oligos, and a PCR target sequence is thus formed. Subsequently, the target sequence is detected and quantified using standard real-time PCR^8^. The 128 plasma samples were distributed randomly on two fluidigm plates with the samples from different time-points from the same patient placed on the same plate. The PEA readout is Normalized Protein Expression (NPX), which is an arbitrary unit on log2 scale where a high NPX value corresponds to a high protein abundance. External and internal controls are included to adjust for intra- and inter-run variation. Assay specific limit of detection is calculated as three times the standard deviation over background signal. Normalization between plates was performed using intensity normalization. Intensity normalization adjusts the data so the median NPX for a protein on each plate is equal to the overall median. Each plate is adjusted so that the median of all assays is the same on all plates.

### Statistical analyses

The statistical analyses conducted in this study on the biomedical multiplex data have two main workflows as seen in Fig. 1. The left side of the flowchart in Fig. 1 shows the workflow for identifying differentially expressed proteins and assessing the potential and accuracy of these proteins to predict whether a sample is from the acute or the stable phase of STEMI. These analyses are described in more detail in the sections “Differentially Expressed Proteins” and “Identification of Predictive Biomarkers and Assessment of Importance”. The right side of the flowchart in Fig. 1 shows the workflow for exploring the response profiles for the investigated proteins. This analysis is described in more detail in the section “Response Profile Analysis”.

### Differentially expressed proteins

A paired t-test was used to test for differential expression of proteins between the acute and the stable phase. The normality assumption of the paired t-test was investigated for the difference between the two tested groups using the Shapiro–Wilk test. If the normality assumption was not met, a Wilcoxon signed rank test was conducted instead. P-values from the tests for differential expression levels were corrected for multiple testing using the Benjamini–Hochberg method^9^. Fold changes were calculated for all statistically significant differentially expressed proteins by transforming data to a linear scale and dividing the mean of the group from the stable phase with the mean of the group from the acute phase for each protein.

### Identification of predictive biomarkers and assessment of importance

In this study, a random forest model was trained on a discovery set of 64 samples from the acute phase (n = 32) and the stable phase (n = 32) originating from 32 patients. The 32 patients were randomly selected among a total of 48 patients. The remaining 16 patients and the corresponding 32 samples from the acute phase (n = 16) and the stable phase (n = 16) were used as a validation set for the trained random forest model.

Proteins with significantly differential expression between the acute and the stable phase were used as predictors in the trained random forest model. The trained random forest model was trained with 500 bootstrapped data sets. The model trained on the discovery set was subsequently assessed based on how correct the model predicts whether a sample from the validation set belongs to the acute or the stable phase. When training the random forest model for each split, the model was only allowed to choose from a small subset of predictors. The numerical magnitude of the small random subset was defined as the square root of the number of predictors in the model^1^. Furthermore, the importance of the protein predictors used in the random forest model, trained using the discovery set, was assessed using the mean decrease of the Gini index for each protein predictor^1^.

The random forest model is based on decision trees. A decision tree classifies objects by segmenting a given predictor space into simple regions using a set of defined splitting rules. A common way to define splitting rules is selecting the split that minimizes the Gini index. The Gini index is a measure of the total variance across the different classes and also referred to as “purity of a region” because a small value of the Gini index indicates that a region contains predominantly observations from a single class^1^ is that decision trees have a high variance. Thus, if a data set was randomly split in two halves and a decision tree was fitted to both halves, the resulting predictions could be quite different. One general approach to reducing the variance of a statistical learning model is called bagging. The bagging procedure reduces the variance by taking the average or the most common prediction generated by many decision trees as the final prediction for an observation^1^.

The initial step of the bagging method is bootstrapping, which generates many different data sets from one original data set. Bootstrapping generates new training data sets of the same size as the original data set that can contain multiple numbers of the same sample. Because of this, bootstrapped data sets resemble the original data set, but with unique differences for each of the new bootstrapped data sets. The next step is to fit decision trees to each of these new training data sets. In the context of classification, each of these fitted decision trees can now be used for predicting the class of an observation. For each observation this will lead to multiple classifications. The most common type of classification among the multiple classifications is selected as the final classification for the given observation. This procedure reduces the variance of the overall predictions and increases the predictive accuracy.

However, highly correlated trees can occur when using the bagging procedure for decision trees. Averaging highly correlated trees will not lead to as large a reduction of the variance as averaging many uncorrelated trees. This problem can be alleviated by using the random forest method. The random forest method still uses the bootstrapping principle to generate many data sets and fit decision trees to each of these data sets. The key difference of the random forest method is that for all splits, the algorithm is only allowed to choose between a small random set of the predictors. This decorrelates with the many decision trees and reduces the variance which gives a higher predictive accuracy. Another aspect of the bagging and random forest method is that the fitted model can be used to assess the importance of a given predictor. This can be done by adding up the total amount that the Gini index is decreased by splits over a given predictor, averaged over all the new trees built from the bootstrap data^1^.

### Response profile analysis

Four samples were collected from 16 patients in the acute phase, 4 h after PPCI, 12 h after PPCI and after three months in the stable phase. This data set will be referred to as the time-series data in subsequent analyses.

The time-series data were used to explore if any of the proteins investigated had similar response profiles over the four time-points. Repeated ANOVA tests were conducted to find proteins where at least one of the four time-point groups were different from at least one of the other time-point groups. The data investigated with the ANOVA test were tested for normality and homogeneity of variance and only moderate departures from normality were found. As the ANOVA test is robust against moderate departures from the assumption of normality, this was not considered problematic. Proteins that obtained significant p-values, after correction for multiple testing were chosen for further analysis using the k-means clustering algorithm. The silhouette method^10^ was used to obtain an estimate for the number of clustering groups used in the k-means clustering algorithm. For each of the chosen proteins, the mean of each of the four time-points in the time-series was calculated and scaled. Scaling was carried out in the following way; for each protein, each of the four time-points values were subtracted by the mean of the four time-point values and divided with the standard deviation of the four time-point values.

A distance matrix between all pairs of the chosen proteins was made using Euclidean distances between the means of the time-points as the distance measure. The k-means clustering algorithm^3^ was used with the distance matrix, thus partitioning the proteins into clustering groups.

### Software

The figures presented in this article are all made with the R software package called ggplot2, version 3.3.2; Wickham. ggplot2: Elegant Graphics for Data Analysis. Springer-Verlag New York, 2016. https://ggplot2.tidyverse.org. The R packages used include *randomForest* for model building, *nbclust* for determining groups of clusters and *kmeans* for clustering of response profiles.

## Results

Out of 92 proteins, 91 were used for further analyses. The abundance level of the protein CCL22 was not used further analysed due to technical issues from the manufacturer.

This article presents two ways of using the multiplex protein data for patients with STEMI. First, data were analysed using the 48 samples from the acute and the stable phase and focused on identifying potential biomarkers testing differential expression and using the statistically significant differentially expressed proteins as predictors in a random forest model (Fig. 1).

The second way of analysing data used the time-series data consisting of 16 samples from each of the groups; in the acute phase, at four hours, 12 h and in the stable phase adding up to 64 samples in total. This way of analysing data focuses on using the k-means clustering algorithm to group the response profiles of the selected proteins over the four time-points (Fig. 1).

**Figure 2:**
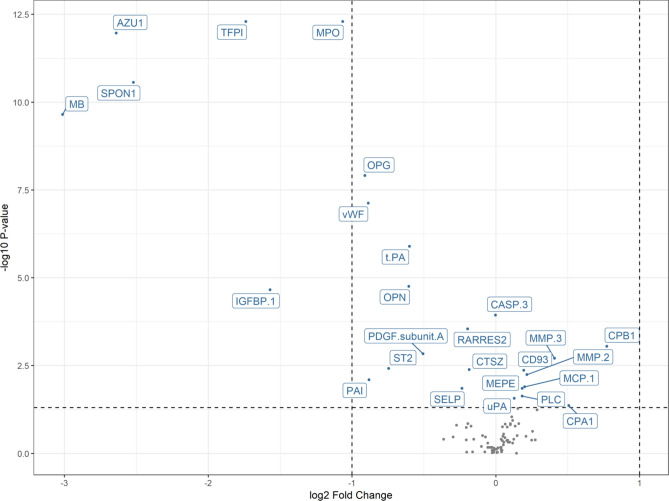
Volcano plot describing the relation between − log10 P-values on the y-axis and log2 fold changes on the x-axis for the 91 proteins. Significant p-values corrected for multiple testing (p < 0.05) are labelled with abbreviated names of the corresponding protein. Horizontal dashed line represents adjusted p-value = 0.05. From left to right, the two vertical dashed lines represent -2 and twofold changes, respectively. This figure was made with the R software package called ggplot2, version 3.3.2: Wickham; ggplot2: Elegant Graphics for Data Analysis. Springer-Verlag New York, 2016: https://ggplot2.tidyverse.org.

A total of 26 out of the 91 proteins were identified as having statistically significantly differential abundance levels between the acute phase of STEMI and the stable phase after correcting for multiple testing using the Benjamini–Hochberg method^9^. All tests for differential expression can be found in Table A2 in the Supplemental Material. A total of nine out of the 26 differentially expressed proteins were found to be up-regulated in the stable phase compared to the acute phase; 17 out of the 26 proteins with differentially expressed proteins were found to be down-regulated in the stable phase compared to the acute phase. These results are presented as a volcano plot describing the relationship between -log10 P-values on the y-axis and log2 fold changes on the x-axis for the 91 proteins (Fig. 2).

**Table 2:** Confusion matrix for the prediction of the status of the 32 samples from the validation set, which were randomly selected.

	Predicted acute phase	Predicted stable phase	Class error rate
True acute phase	15	1	0.065
True stable phase	0	16	0

The table shows the results of training the random forest model on the discovery set and using the trained model to predict the sample status of samples included in the validation set.

A random forest model was trained using the discovery set consisting of 32 patients, randomly selected among 48 patients, and the corresponding 64 samples from the acute phase (n = 32) and the stable phase (n = 32). The remaining 16 patients and the corresponding 32 samples from the acute phase (n = 16) and the stable phase (n = 16) were used as a validation set. This fitted random forest model was used to predict the status of 32 samples from the validation set, which can be seen in Table 2. Out of the 16 samples from the acute phase, 15 were predicted correctly, which gives a class error rate of 0.06. All of the 16 samples from the stable phase were predicted correctly. Out of 32 samples, 31 samples were predicted correctly, resulting in an overall error rate of 0.04.

The random forest model trained using the discovery set was used to determine the importance of the 26 protein predictors by using the mean decrease of the Gini index. A plot of the predictor importance for the random forest model can be seen in Fig. 2. The plot shows the importance of the 26 protein biomarker predictors plotted as the mean decrease of the Gini index relative to the protein tissue factor pathway inhibitor (TFPI). This was done because the protein TFPI had the highest mean decrease of the Gini index in the random forest model.

The time-series data were used to investigate if any of the 91 proteins had similar response profiles over the four time-points. The first step of the response profile analysis was to use repeated measure ANOVA, which identified a total of 53 significant proteins after correction for multiple testing^9^ All ANOVA tests are shown in Table A3 in the Supplemental Material. The means of the four time-points were calculated for each of the 53 proteins and the means of the time-groups for each protein were then used to determine the estimated number of clustering groups using the silhouette method^10^ (Supplemental Material Figure A1). The silhouette method indicates that the number of clusters is seven (Supplemental Material Figure A1). The means of the four time-groups for each protein were then clustered together using the k-means clustering algorithm with an estimate of seven clustering groups. This information was visualized using scaled boxplots for each of the proteins in a clustering group.

Clustering group 1 (Fig. 4) and clustering group 6 (Fig. 5) were chosen after inspecting the seven clustering groups, because these two groups showed the most uniform response profile pattern. Plots of the five remaining clustering groups can be found in Figures A2–A6 in the supplemental material.

## Discussion

The present study demonstrates how advanced statistical and machine learning methods can be used for analysing multiplex protein data obtained from a novel proximity extension assay. A study by Kulasingam et al. used a qualitative approach to analyse the multiplex protein data. This more conventional approach focused on testing differential expression between the acute and the stable phase. Furthermore, in the study by Kulasingam et al., the fold changes of the differentially expressed proteins were calculated, and the differentially expressed proteins were manually grouped according to the associated general molecular functions. The present study supplements the previous qualitative approach^11^ by analysing additional samples with focus on exploring the use of quantitative methods based on advanced statistical and machine learning analyses.

In particular, it was found that 26 proteins were differentially expressed between the acute phase of STEMI and the stable phase three months later. Out of these 26 proteins, the proteins tissue factor pathway inhibitor, azurocidin, spondin-1, myeloperoxidase and myoglobin had very low p-values and large negative fold changes (Fig. 2) indicating a large decrease in the abundance level of these five proteins between the acute and the stable phase.

It was hypothesised that a smaller fraction of the 26 proteins was highly important in the prediction of whether a sample belonged to the acute or the stable phase. Thus, a random forest model was trained using a discovery set consisting of 64 samples from the acute phase and the stable phase using the 26 significant proteins as predictors. The trained random forest model was used to predict which group, acute or stable phase, the 32 samples from the validation set belonged to. The trained model had an overall error rate of 0.04. The random forest model showed a little higher error rate for predicting the acute phase than the stable phase, suggesting that the samples from the acute phase are more diverse regarding the expression of the 26 protein predictors. This may suggest that the pathological process occurring during the acute phase is more variable and unpredictable than the molecular processes in the stable phase. However, as the class error rate for the acute phase is relatively small, the predictions for this class are considered within the range of accurate prediction.

**Figure 3:**
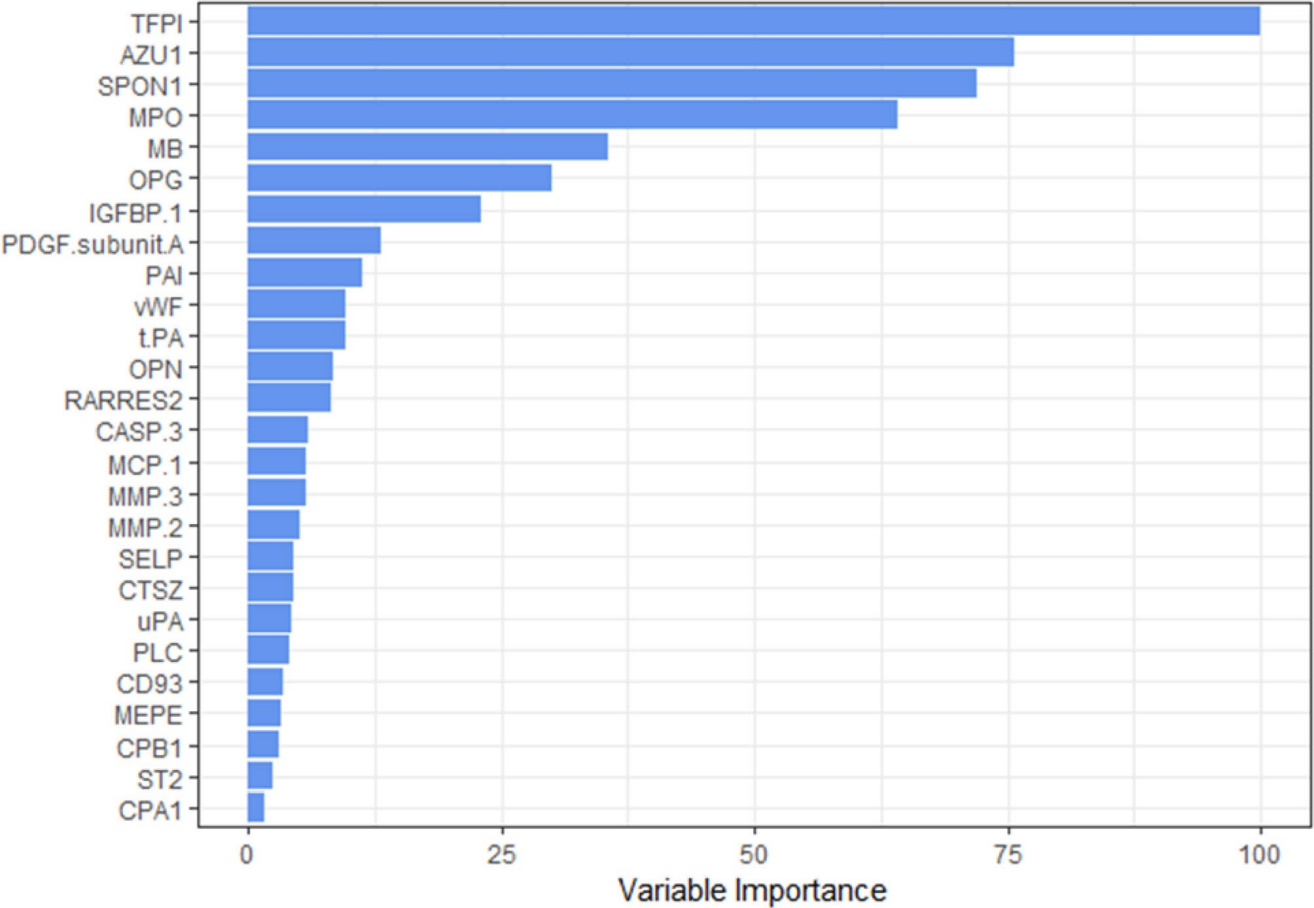
Predictor importance plot for the random forest model trained on the discovery set. Predictor importance is computed using the mean decrease in Gini index, and plotted relative to the protein tissue factor pathway inhibitor (TFPI), which had the maximum mean decrease in Gini index among the 26 proteins. This figure was made with the R software package called ggplot2, version 3.3.2: Wickham; ggplot2: Elegant Graphics for Data Analysis. Springer-Verlag New York, 2016: https://ggplot2.tidyverse.org.

These results suggest that the protein predictors included in the random forest model have a high predictive accuracy. The five proteins tissue factor pathway inhibitor, azurocidin, spondin-1, myeloperoxidase and myoglobin were found to be highly important for distinguishing between the acute phase of STEMI and the stable phase (Fig. 3) assessed by the random forest model. The results indicate that the protein tissue factor pathway inhibitor is a particularly strong predictor for describing the pathological differences between the acute and the stable phase of STEMI.

The protein abundance level of tissue factor pathway inhibitor were found to be strongly decreased from the acute phase to the stable phase. Similar results were found in a study by Winckers et al. (2011)^11^ showing that healthy young women without myocardial infarction had lower levels of tissue factor pathway inhibitor than young women with myocardial infarction^11^.

When choosing a model for predictive analysis, it is important to keep in mind that most predictive models work under certain assumptions about the data. For example, if a linear relationship exists between the predictors and the predicted variable, linear regression models tend to have higher predictive accuracy than decision trees^1^. When analysing many variables such as biomarker proteins, one could imagine the existence of non-linear relationships between the protein predictors and the predicted variable of interest. In general, the random forest model and decision trees are less strict with prior assumptions of specific kinds of relationships between the predictors and the predicted variable. Hence, the random forest model and models based on decision trees are both suitable approaches for development of predictive models. The models developed may identify novel promising biomarker candidates with the potential to predict for example diagnosis, a clinical outcome or stratify patients to a specific treatment. It should be mentioned that the random forest model can perform in unexpected ways if a large number of the predictors included in the model are highly correlated. In biological data sets, like multiplex protein data investigated here, some of the proteins are expected to be correlated, which means that the biological predictors included in the model are correlated. According to Tibshirani et al. 2013, this problem can be alleviated by letting the random forest model choose from a small random set of all the predictors when a split is generated. The square root of the number of predictors has been shown to work well for choosing the numerical magnitude of a small random set of predictors^1^. Another approach to investigating and reducing the collinearity among the predictors in a data set could be to use principal component analysis^4^ to reduce the dimensions of the data. If only a small number of the corresponding principal components describe a large amount of the variability in the data set, these principal components could be used as predictors in the random forest model and thus reduce the collinearity of the data set. However, from a clinical or biological point of view, this principal component analysis procedure might not be favoured as it also reduces the possibility of any direct biological or clinical interpretation of the predictors used in the random forest model.

**Figure 4:**
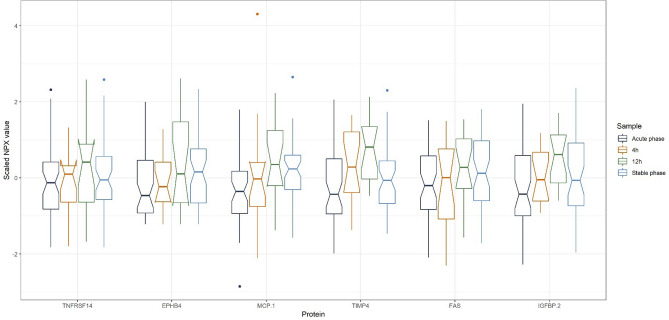
Box plots for the time-points acute phase, 4 h, 12 h, and stable phase for the six proteins in clustering group 1. Notches determines the 95% confidence interval for the median value. On the y-axis scaled NPX values are shown. The scaled NPX values cannot be compared directly between proteins, but only between measurements of the same protein. This figure was made with the R software package called ggplot2, version 3.3.2: Wickham; ggplot2: Elegant Graphics for Data Analysis. Springer-Verlag New York, 2016: https://ggplot2.tidyverse.org.

**Figure 5:**
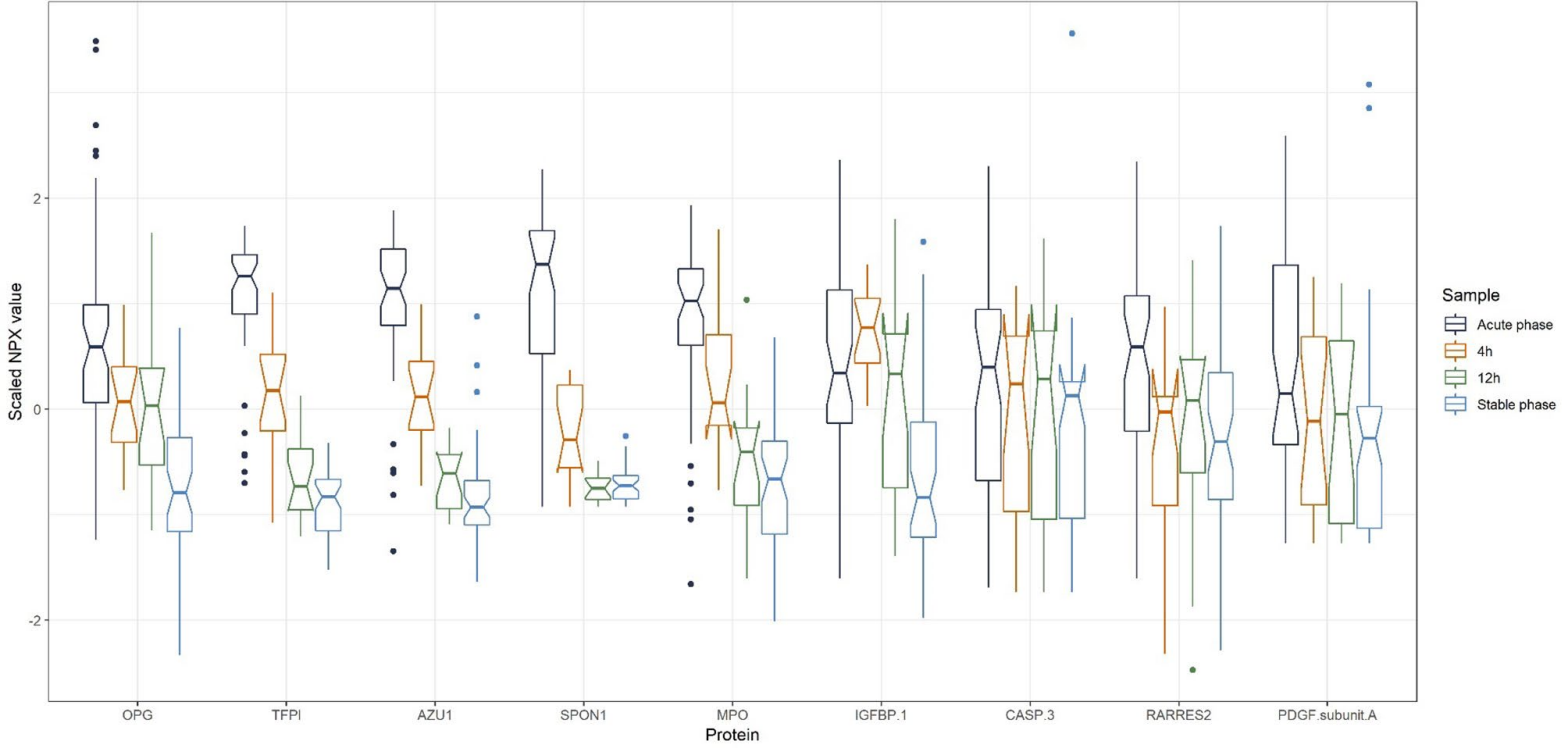
Box plots for the time-points acute phase, 4 h, 12 h, and stable phase for the nine proteins in clustering group 6. Notches determine the 95% confidence interval for the median value. On the y-axis scaled NPX values are shown. The scaled NPX values cannot be compared directly between proteins, but only between measurements of the same protein. This figure was made with the R software package called ggplot2, version 3.3.2: Wickham; ggplot2: Elegant Graphics for Data Analysis. Springer-Verlag New York, 2016: https://ggplot2.tidyverse.org.

To investigate if any of the 91 protein biomarkers showed similar response profiles, a k-means clustering algorithm was used to cluster the means of the four time-groups for each of the 91 proteins. This resulted in seven clustering groups, among which group 1 (Fig. 4) and group 6 (Fig. 5) were specifically interesting as they exhibited uniform response profiles within the clustering group. The clustering groups might point out proteins with similar response profiles over the four time-points and could point out proteins that might be collinear. Candidates for proteins that might share similar response profiles could be the proteins tissue factor pathway inhibitor, azurocidin, spondin-1 and myeloperoxidase from group 6, as these proteins exhibit very similar response profiles and thus might also be colinear. As these four proteins are found to be important predictors in the random forest model this might mean, if these protein predictors are colinear, that these four proteins contribute with the same or similar information to distinguish between the acute and the stable phase.

The high predictive accuracy of the 26 proteins used as predictors in the random forest model and the assessment of importance of the five proteins tissue factor pathway inhibitor, azurocidin, spondin-1, myeloperoxidase and myoglobin may indicate that these five proteins have important roles for the pathogenesis and/or the healing process in STEMI patients. The five proteins tissue factor pathway inhibitor, azurocidin, spondin-1, myeloperoxidase and myoglobin are known to be associated with antithrombotic and antibacterial activity, tissue damage, muscle injury, inflammation and cytokine production^12–17^. All of these processes are highly relevant for the acute phase of STEMI, thus corroborating the finding that these proteins are able to differentiate between the acute and the stable phase of STEMI.

More studies are warranted to investigate if tissue factor pathway inhibitor, azurocidin, spondin-1, myeloperoxidase and myoglobin have an important role in STEMI and if they may be used as biomarkers associated with STEMI. Elements of further analyses could include collecting samples from a large discovery cohort and a large validation cohort and including analysis of healthy control samples. Including data from healthy control samples could indicate whether the stable phase of STEMI resembles or are similar to normal healthy molecular processes of the proteins investigated.

The data analysis approach presented in this study is also feasible in other areas employing large biomarker data sets, like miRNA^18^ or mRNA analysis^19^. Moreover, the presented data analysis workflow can also be used with other technologies generating multiplex data and is not limited to any specific disease or condition^18,19^.

The study is limited by the small sample size challenging the possibility of performing multiple adjustments for e.g. sex, age or BMI (Table2).

## Conclusion and perspectives

Using a random forest model, the protein biomarkers tissue factor pathway inhibitor, azurocidin, spondin-1, myeloperoxidase and myoglobin were found to have high importance for describing the pathological differences between the acute phase and stable phase for patients with STEMI. Thus, these five proteins are good candidates for further studies investigating biomarkers for healing processes in STEMI patients, and can contribute to an in-depth understanding of the molecular processes occurring from PPCI to three months follow-up. Furthermore, by using a k-means clustering algorithm, it was found that tissue factor pathway inhibitor, azurocidin, spondin-1 and myeloperoxidase seem to have similar response profiles over the acute phase, four hours after PPCI, 12 h after PPCI and in the stable phase three months later. The presented advanced statistical and machine learning methods are widely applicable to biological data with multiplex dimensions. The advanced statistical and machine learning methods are not to any specific technology, disease or condition, but as suggested by the present data, these methods could for example be implemented as software available to the staff conducting the prehospital diagnosis of STEMI and NSTEMI.

In future studies, the comparison of different machine learning methods such as random forest, LASSO regression or the more standard logistic regression should be done to obtain more knowledge on the benefit of different statistical approaches. Furthermore, future studies should be designed to enable external validation of the machine learning methods in other patient populations.

## Supplementary Information

Supplementary Information.

## Data Availability

The datasets and coding employed in the present study are available upon reasonable request to the first author of the manuscript.
