# Supplementary material for: Statistical and machine learning methods for analysis of multiplex protein data from a novel proximity extension assay in patients with ST-elevation myocardial infarction
Source: Sci Rep. 2021 Jul 2;11:13787. doi: 10.1038/s41598-021-93162-3 (PMC8253786; doi:10.1038/s41598-021-93162-3)
Supplement: Supplementary file 1 — Supplementary Information. [file 41598_2021_93162_MOESM1_ESM.docx]

# Supplementary Material

**Statistical and Machine Learning Methods for Analysis of Multiplex Protein Data from a Novel Proximity Extension Assay in patients with ST-elevation myocardial infarction**

**Authors:**

Emil Maag^a b^, Archana Kulasingam^c^, Erik Lerkevang Grove^c d^, Kamilla Sofie Pedersen^a^, Steen Dalby Kristensen^c d^, Anne-Mette Hvas^d e^

**Affiliations**

Emil Maag, phone +45 20749325, emil@bioxpedia.com.

Archana Kulasingam, phone +45 20685181, archana.kulasingam@rsyd.dk

Erik Lerkevang Grove, phone +45 40142993, erikgrove@dadlnet.dk.

Kamilla Sofie Pedersen, phone +45 28199690, kamilla.sofie@gmail.com.

Steen Dalby Kristensen, phone +45 30922336, steendk@dadlnet.dk

Anne-Mette Hvas, phone +45 21186893, am.hvas@dadlnet.dk

**Corresponding author**: Erik Lerkevang Grove, email [erikgrove@dadlnet.dk](mailto:erikgrove@dadlnet.dk).

^a^ BioXpedia A/S, Palle Juul-Jensens Blvd 82, 8200 Aarhus N, Denmark

^b^ Bioinformatics Research Centre (BiRC), Aarhus University, C.F. Møllers Allé 8, Building 110, 8000 Aarhus C, Denmark

^c^ Department of Cardiology, Aarhus University Hospital, Palle Juul-Jensens Blvd 99, 8200 Aarhus N, Denmark

^d^ Department of Clinical Medicine, Faculty of Health, Aarhus University, Palle Juul-Jensens Blvd 82, 8200 Aarhus N, Denmark

^e^ Thrombosis & Haemostasis Research Unit, Department of Clinical Biochemistry, Aarhus University Hospital, Palle Juul-Jensens Blvd 99, 8200 Aarhus N, Denmark

# Table A1. Abbreviations, Uniprot Id and full protein name of the 92 proteins investigated in this study, from the biomarker panel CARDIOVASCULAR III (Olink^®^ Bioscience, Uppsala, Sweden).

| Abbreviation | Uniprot Id | Full protein name |
| --- | --- | --- |
| ALCAM | Q13740 | CD166 antigen |
| AP.N | P15144 | Aminopeptidase N |
| AXL | P30530 | Tyrosine-protein kinase receptor UFO |
| AZU1 | P20160 | Azurocidin |
| BLM.hydrolase | Q13867 | Bleomycin hydrolase |
| CASP.3 | P42574 | Caspase-3 |
| CCL15 | Q16663 | C-C motif chemokine 15 |
| CCL16 | O15467 | C-C motif chemokine 16 |
| CCL24 | O00175 | C-C motif chemokine 24 |
| CD163 | Q86VB7 | Scavenger receptor cysteine-rich type 1 protein M130 |
| CD93 | Q9NPY3 | Complement component C1q receptor |
| CDH5 | P33151 | Cadherin-5 |
| CHI3L1 | P36222 | Chitinase-3-like protein 1 |
| CHIT1 | Q13231 | Chitotriosidase-1 |
| CNTN1 | Q12860 | Contactin-1 |
| COL1A1 | P02452 | Collagen alpha-1(I) chain |
| CPA1 | P15085 | Carboxypeptidase A1 |
| CPB1 | P15086 | Carboxypeptidase B |
| CSTB | P04080 | Cystatin-B |
| CTSD | P07339 | Cathepsin D |
| CTSZ | Q9UBR2 | Cathepsin Z |
| CXCL16 | Q9H2A7 | C-X-C motif chemokine 16 |
| DLK.1 | P80370 | Protein delta homolog 1 |
| EGFR | P00533 | Epidermal growth factor receptor |
| Ep.CAM | P16422 | Epithelial cell adhesion molecule |
| EPHB4 | P54760 | Ephrin type-B receptor 4 |
| FABP4 | P15090 | Fatty acid-binding protein, adipocyte |
| FAS | P25445 | Tumor necrosis factor receptor superfamily member 6 |
| Gal.3 | P17931 | Galectin-3 |
| Gal.4 | P56470 | Galectin-4 |
| GDF.15 | Q99988 | Growth differentiation factor 15 |
| GRN | P28799 | Granulins |
| ICAM.2 | P13598 | Intercellular adhesion molecule 2 |
| IGFBP.1 | P08833 | Insulin-like growth factor-binding protein 1 |
| IGFBP.2 | P18065 | Insulin-like growth factor-binding protein 2 |
| IGFBP.7 | Q16270 | Insulin-like growth factor-binding protein 7 |
| IL.17RA | Q96F46 | Interleukin-17 receptor A |
| IL.18BP | O95998 | Interleukin-18 binding protein |
| IL.1RT1 | P14778 | Interleukin-1 receptor type 1 |
| IL.1RT2 | P27930 | Interleukin-1 receptor type 2 |
| IL2.RA | P01589 | Interleukin-2 receptor subunit alpha |
| IL6.RA | P08887 | Interleukin-6 receptor subunit alpha |
| ITGB2 | P05107 | Integrin beta-2 |
| JAM.A | Q9Y624 | Junctional adhesion molecule A |
| KLK6 | Q92876 | Kallikrein-6 |
| LDL.receptor | P01130 | Low-density lipoprotein receptor |
| LTBR | P36941 | Lymphotoxin-beta receptor |
| MB | P02144 | Myoglobin |
| MCP.1 | P13500 | Monocyte chemotactic protein 1 |
| MEPE | Q9NQ76 | Matrix extracellular phosphoglycoprotein |
| MMP.2 | P08253 | Matrix metalloproteinase-2 |
| MMP.3 | P08254 | Matrix metalloproteinase-3 |
| MMP.9 | P14780 | Matrix metalloproteinase-9 |
| MPO | P05164 | Myeloperoxidase |
| Notch 3 | Q9UM47 | Neurogenic locus notch homolog protein 3 |
| NT.proBNP | NA | N-terminal prohormone brain natriuretic peptide |
| OPG | O00300 | Osteoprotegerin |
| OPN | P10451 | Osteopontin |
| PAI | P05121 | Plasminogen activator inhibitor 1 |
| PCSK9 | Q8NBP7 | Proprotein convertase subtilisin/kexin type 9 |
| PDGF.subunit.A | P04085 | Platelet-derived growth factor subunit A |
| PECAM.1 | P16284 | Platelet and endothelial cell adhesion molecule 1 |
| PGLYRP1 | O75594 | Peptidoglycan recognition protein 1 |
| PI3 | P19957 | Elafin |
| PLC | P98160 | Perlecan |
| PON3 | Q15166 | Paraoxonase 3 |
| PRTN3 | P24158 | Myeloblastin |
| PSP.D | P35247 | Pulmonary surfactant-associated protein D |
| RARRES2 | Q99969 | Retinoic acid receptor responder protein 2 |
| RETN | Q9HD89 | Resistin |
| SCGB3A2 | Q96PL1 | Secretoglobin family 3A member 2 |
| SELE | P16581 | E-selectin |
| SELP | P16109 | P-selectin |
| SHPS.1 | P78324 | Tyrosine-protein phosphatase non-receptor type substrate 1 |
| SPON1 | Q9HCB6 | Spondin-1 |
| ST2 | Q01638 | ST2 protein |
| t.PA | Q07654 | Tissue-type plasminogen activator |
| TFF3 | P10646 | Trefoil factor 3 |
| TFPI | Q99727 | Tissue factor pathway inhibitor |
| TIMP4 | Q5T2D2 | Metalloproteinase inhibitor 4 |
| TLT.2 | P19438 | Trem-like transcript 2 protein |
| TNF.R1 | P20333 | Tumor necrosis factor receptor 1 |
| TNF.R2 | O14798 | Tumor necrosis factor receptor 2 |
| TNFRSF10C | Q92956 | Tumor necrosis factor receptor superfamily member 10C |
| TNFRSF14 | Q9Y275 | Tumor necrosis factor receptor superfamily member 14 |
| TNFSF13B | P00750 | Tumor necrosis factor ligand superfamily member 13B |
| TR | P02786 | Transferrin receptor protein 1 |
| TR.AP | P13686 | Tartrate-resistant acid phosphatase type 5 |
| U.PAR | P00749 | Urokinase plasminogen activator surface receptor |
| uPA | Q03405 | Urokinase-type plasminogen activator |
| vWF | P04275 | von Willebrand factor |
| CCL22 | O00626 | C-C motif chemokine 22 |

Table A2. Results for testing differential expression between the acute phase and the stable phase of STEMI. The table lists the abbreviated names of the proteins, the p-value, the fold change and the adjusted p-value (Benjamini & Hochberg, 1995).

| **Protein** | **P-value** | **Fold Changes** | **Adjusted P-value** |
| --- | --- | --- | --- |
| TFPI | 7.11E-15 | 1.21E+00 | 6.47E-13 |
| AZU1 | 3.55E-14 | 2.06E+00 | 1.62E-12 |
| SPON1 | 1.80E-12 | 5.04E+00 | 5.45E-11 |
| MPO | 4.55E-12 | 1.36E+00 | 1.03E-10 |
| MB | 4.08E-11 | 1.43E+00 | 7.42E-10 |
| OPG | 1.25E-09 | 1.37E+00 | 1.90E-08 |
| vWF | 7.47E-09 | 1.16E+00 | 9.72E-08 |
| IGFBP.1 | 5.28E-07 | 1.69E+00 | 6.01E-06 |
| OPN | 1.33E-06 | 1.17E+00 | 1.35E-05 |
| t.PA | 1.63E-06 | 1.10E+00 | 1.48E-05 |
| CASP.3 | 2.07E-05 | 1.17E+00 | 1.72E-04 |
| RARRES2 | 3.82E-05 | 1.02E+00 | 2.90E-04 |
| CPB1 | 1.15E-04 | 8.38E-01 | 8.06E-04 |
| PDGF.subunit.A | 1.86E-04 | 2.04E+00 | 1.21E-03 |
| MMP.3 | 3.80E-04 | 9.16E-01 | 2.18E-03 |
| PAI | 3.84E-04 | 1.23E+00 | 2.18E-03 |
| CTSZ | 5.66E-04 | 1.04E+00 | 3.03E-03 |
| ST2 | 1.14E-03 | 1.17E+00 | 5.76E-03 |
| CD93 | 1.55E-03 | 9.78E-01 | 7.42E-03 |
| MCP.1 | 2.74E-03 | 9.21E-01 | 1.25E-02 |
| SELP | 3.28E-03 | 1.04E+00 | 1.42E-02 |
| MEPE | 4.11E-03 | 8.84E-01 | 1.70E-02 |
| MMP.2 | 5.50E-03 | 9.34E-01 | 2.17E-02 |
| uPA | 6.60E-03 | 9.67E-01 | 2.50E-02 |
| CPA1 | 9.31E-03 | 9.25E-01 | 3.39E-02 |
| PLC | 1.05E-02 | 9.63E-01 | 3.69E-02 |
| CDH5 | 2.28E-02 | 9.31E-01 | 7.68E-02 |
| GDF.15 | 2.45E-02 | 1.06E+00 | 7.97E-02 |
| IGFBP.2 | 4.02E-02 | 9.60E-01 | 1.22E-01 |
| ALCAM | 3.97E-02 | 9.77E-01 | 1.22E-01 |
| CTSD | 4.56E-02 | 1.03E+00 | 1.34E-01 |
| ITGB2 | 5.56E-02 | 9.73E-01 | 1.58E-01 |
| EPHB4 | 5.79E-02 | 9.32E-01 | 1.60E-01 |
| FAS | 6.12E-02 | 9.74E-01 | 1.64E-01 |
| PRTN3 | 7.00E-02 | 1.04E+00 | 1.82E-01 |
| AXL | 7.78E-02 | 9.82E-01 | 1.97E-01 |
| IL2.RA | 8.74E-02 | 9.46E-01 | 2.15E-01 |
| MMP.9 | 8.97E-02 | 1.12E+00 | 2.15E-01 |
| NT.proBNP | 9.42E-02 | 7.40E-01 | 2.20E-01 |
| TNF.R2 | 1.04E-01 | 9.83E-01 | 2.38E-01 |
| TIMP4 | 1.14E-01 | 9.42E-01 | 2.52E-01 |
| COL1A1 | 1.17E-01 | 9.56E-01 | 2.53E-01 |
| TR.AP | 1.28E-01 | 1.02E+00 | 2.70E-01 |
| TNFSF13B | 1.39E-01 | 9.81E-01 | 2.88E-01 |
| PON3 | 1.52E-01 | 1.03E+00 | 3.02E-01 |
| Notch.3 | 1.52E-01 | 9.40E-01 | 3.02E-01 |
| IL.18BP | 1.57E-01 | 9.82E-01 | 3.03E-01 |
| SCGB3A2 | 1.60E-01 | 1.16E+00 | 3.03E-01 |
| LDL.receptor | 1.69E-01 | 1.06E+00 | 3.13E-01 |
| CXCL16 | 2.21E-01 | 1.02E+00 | 4.02E-01 |
| PCSK9 | 2.28E-01 | 8.94E-01 | 4.07E-01 |
| PI3 | 2.64E-01 | 9.55E-01 | 4.14E-01 |
| CHIT1 | 2.55E-01 | 9.61E-01 | 4.14E-01 |
| DLK.1 | 2.53E-01 | 9.70E-01 | 4.14E-01 |
| TLT.2 | 2.54E-01 | 9.65E-01 | 4.14E-01 |
| CNTN1 | 2.46E-01 | 9.64E-01 | 4.14E-01 |
| BLM.hydrolase | 2.61E-01 | 9.87E-01 | 4.14E-01 |
| GRN | 2.55E-01 | 9.86E-01 | 4.14E-01 |
| CCL15 | 3.01E-01 | 9.87E-01 | 4.57E-01 |
| TNFRSF14 | 3.01E-01 | 9.76E-01 | 4.57E-01 |
| JAM.A | 3.41E-01 | 1.03E+00 | 5.01E-01 |
| IL.6RA | 3.41E-01 | 9.94E-01 | 5.01E-01 |
| CCL24 | 3.78E-01 | 1.03E+00 | 5.46E-01 |
| TNF.R1 | 4.36E-01 | 1.00E+00 | 6.11E-01 |
| SELE | 4.36E-01 | 1.05E+00 | 6.11E-01 |
| AP.N | 4.79E-01 | 9.93E-01 | 6.51E-01 |
| CD163 | 4.78E-01 | 9.89E-01 | 6.51E-01 |
| PECAM.1 | 4.99E-01 | 1.02E+00 | 6.67E-01 |
| TR | 5.21E-01 | 1.02E+00 | 6.87E-01 |
| EGFR | 5.52E-01 | 1.01E+00 | 7.10E-01 |
| Gal.3 | 5.59E-01 | 9.90E-01 | 7.10E-01 |
| TFF3 | 5.61E-01 | 9.91E-01 | 7.10E-01 |
| IGFBP.7 | 6.00E-01 | 9.99E-01 | 7.49E-01 |
| CCL16 | 6.25E-01 | 1.01E+00 | 7.58E-01 |
| SHPS.1 | 6.36E-01 | 9.83E-01 | 7.58E-01 |
| IL.1RT2 | 6.35E-01 | 1.01E+00 | 7.58E-01 |
| PSP.D | 6.51E-01 | 9.58E-01 | 7.58E-01 |
| LTBR | 6.57E-01 | 9.84E-01 | 7.58E-01 |
| CSTB | 6.58E-01 | 1.02E+00 | 7.58E-01 |
| ICAM.2 | 6.75E-01 | 9.92E-01 | 7.68E-01 |
| TNFRSF10C | 7.48E-01 | 1.01E+00 | 8.41E-01 |
| RETN | 7.61E-01 | 1.01E+00 | 8.45E-01 |
| U.PAR | 7.89E-01 | 1.01E+00 | 8.57E-01 |
| IL.17RA | 7.91E-01 | 9.88E-01 | 8.57E-01 |
| PGLYRP1 | 8.20E-01 | 9.97E-01 | 8.62E-01 |
| IL.1RT1 | 8.24E-01 | 1.00E+00 | 8.62E-01 |
| Ep.CAM | 8.11E-01 | 9.90E-01 | 8.62E-01 |
| FABP4 | 8.99E-01 | 1.01E+00 | 9.30E-01 |
| KLK6 | 9.64E-01 | 9.99E-01 | 9.74E-01 |
| Gal.4 | 9.64E-01 | 1.02E+00 | 9.74E-01 |
| CHI3L1 | 9.87E-01 | 1.00E+00 | 9.87E-01 |

Table A3. Results for all repeated measure ANOVA tests. The table lists the abbreviated names of the proteins, the p-value, the F-value and the adjusted p-value (Benjamini & Hochberg, 1995).

| **Protein** | **P-value** | **F-value** | **Adjusted P-value** |
| --- | --- | --- | --- |
| TFPI | 1.46E-31 | 144.73 | 1.33E-29 |
| AZU1 | 2.49E-29 | 123.39 | 1.13E-27 |
| SPON1 | 9.81E-25 | 87.47 | 2.98E-23 |
| MPO | 2.96E-22 | 71.82 | 6.73E-21 |
| OPG | 1.80E-18 | 51.98 | 3.27E-17 |
| MB | 2.12E-15 | 38.87 | 3.22E-14 |
| MMP.3 | 4.52E-14 | 33.90 | 5.88E-13 |
| vWF | 3.12E-13 | 30.95 | 3.55E-12 |
| OPN | 4.26E-13 | 30.49 | 4.31E-12 |
| TIMP4 | 4.28E-12 | 27.19 | 3.89E-11 |
| ST2 | 1.07E-11 | 25.94 | 8.82E-11 |
| t.PA | 1.63E-11 | 25.36 | 1.24E-10 |
| IGFBP.1 | 2.89E-10 | 21.65 | 2.02E-09 |
| CXCL16 | 2.76E-09 | 18.91 | 1.79E-08 |
| PAI | 1.22E-07 | 14.65 | 7.41E-07 |
| IGFBP.2 | 3.80E-07 | 13.46 | 2.15E-06 |
| GDF.15 | 4.02E-07 | 13.40 | 2.15E-06 |
| CTSZ | 5.08E-07 | 13.16 | 2.57E-06 |
| NT.proBNP | 6.53E-07 | 12.90 | 3.13E-06 |
| RARRES2 | 1.25E-06 | 12.24 | 5.67E-06 |
| CPB1 | 1.87E-06 | 11.83 | 8.08E-06 |
| MEPE | 2.15E-06 | 11.69 | 8.70E-06 |
| MMP.2 | 2.20E-06 | 11.67 | 8.70E-06 |
| SCGB3A2 | 3.06E-05 | 9.13 | 1.16E-04 |
| CHI3L1 | 8.97E-05 | 8.14 | 3.26E-04 |
| CTSD | 1.86E-04 | 7.48 | 6.50E-04 |
| SELP | 2.23E-04 | 7.31 | 7.51E-04 |
| PON3 | 2.60E-04 | 7.18 | 8.44E-04 |
| PRTN3 | 3.00E-04 | 7.05 | 9.40E-04 |
| PDGF.subunit.A | 3.15E-04 | 7.01 | 9.54E-04 |
| CD93 | 3.85E-04 | 6.83 | 1.13E-03 |
| TNFRSF10C | 5.02E-04 | 6.59 | 1.43E-03 |
| PLC | 6.49E-04 | 6.37 | 1.79E-03 |
| PI3 | 1.01E-03 | 5.99 | 2.70E-03 |
| MCP.1 | 1.32E-03 | 5.75 | 3.44E-03 |
| IL.1RT1 | 1.43E-03 | 5.69 | 3.55E-03 |
| LDL.receptor | 1.44E-03 | 5.68 | 3.55E-03 |
| CHIT1 | 1.58E-03 | 5.60 | 3.77E-03 |
| IL2.RA | 1.81E-03 | 5.49 | 4.12E-03 |
| CDH5 | 1.81E-03 | 5.49 | 4.12E-03 |
| uPA | 2.47E-03 | 5.22 | 5.49E-03 |
| TNF.R1 | 2.69E-03 | 5.15 | 5.84E-03 |
| EPHB4 | 2.95E-03 | 5.07 | 6.24E-03 |
| CASP.3 | 6.40E-03 | 4.42 | 1.32E-02 |
| TR.AP | 7.34E-03 | 4.30 | 1.48E-02 |
| Notch.3 | 7.68E-03 | 4.27 | 1.52E-02 |
| TLT.2 | 8.85E-03 | 4.15 | 1.71E-02 |
| CPA1 | 9.88E-03 | 4.06 | 1.87E-02 |
| FAS | 1.59E-02 | 3.66 | 2.90E-02 |
| SELE | 1.59E-02 | 3.66 | 2.90E-02 |
| TNFRSF14 | 1.65E-02 | 3.63 | 2.94E-02 |
| CCL15 | 2.01E-02 | 3.47 | 3.51E-02 |
| AXL | 2.69E-02 | 3.23 | 4.62E-02 |
| Gal.3 | 3.43E-02 | 3.03 | 5.79E-02 |
| ALCAM | 3.88E-02 | 2.93 | 6.43E-02 |
| CSTB | 4.16E-02 | 2.87 | 6.77E-02 |
| DLK.1 | 5.46E-02 | 2.65 | 8.71E-02 |
| COL1A1 | 6.72E-02 | 2.48 | 1.04E-01 |
| RETN | 6.83E-02 | 2.47 | 1.04E-01 |
| TFF3 | 6.87E-02 | 2.46 | 1.04E-01 |
| ITGB2 | 7.21E-02 | 2.42 | 1.08E-01 |
| TNF.R2 | 9.27E-02 | 2.22 | 1.36E-01 |
| BLM.hydrolase | 9.75E-02 | 2.18 | 1.41E-01 |
| IL.17RA | 1.05E-01 | 2.12 | 1.49E-01 |
| CCL24 | 1.07E-01 | 2.10 | 1.50E-01 |
| IL.1RT2 | 1.57E-01 | 1.79 | 2.16E-01 |
| TNFSF13B | 1.77E-01 | 1.69 | 2.40E-01 |
| PGLYRP1 | 1.94E-01 | 1.61 | 2.56E-01 |
| MMP.9 | 1.94E-01 | 1.61 | 2.56E-01 |
| IL.6RA | 2.02E-01 | 1.58 | 2.63E-01 |
| GRN | 2.07E-01 | 1.55 | 2.66E-01 |
| Ep.CAM | 2.26E-01 | 1.48 | 2.86E-01 |
| LTBR | 2.70E-01 | 1.33 | 3.37E-01 |
| EGFR | 2.79E-01 | 1.30 | 3.43E-01 |
| CNTN1 | 2.98E-01 | 1.25 | 3.61E-01 |
| FABP4 | 3.11E-01 | 1.21 | 3.68E-01 |
| IL.18BP | 3.12E-01 | 1.21 | 3.68E-01 |
| Gal.4 | 3.24E-01 | 1.18 | 3.78E-01 |
| SHPS.1 | 3.64E-01 | 1.08 | 4.19E-01 |
| KLK6 | 3.86E-01 | 1.03 | 4.39E-01 |
| PSP.D | 3.93E-01 | 1.01 | 4.42E-01 |
| TR | 4.22E-01 | 0.95 | 4.68E-01 |
| CD163 | 4.33E-01 | 0.93 | 4.74E-01 |
| U.PAR | 4.50E-01 | 0.89 | 4.87E-01 |
| CCL16 | 4.55E-01 | 0.88 | 4.87E-01 |
| PCSK9 | 4.70E-01 | 0.85 | 4.97E-01 |
| PECAM.1 | 5.96E-01 | 0.63 | 6.24E-01 |
| IGFBP.7 | 6.25E-01 | 0.59 | 6.46E-01 |
| AP.N | 7.84E-01 | 0.36 | 8.01E-01 |
| JAM.A | 7.95E-01 | 0.34 | 8.04E-01 |
| ICAM.2 | 8.83E-01 | 0.22 | 8.83E-01 |


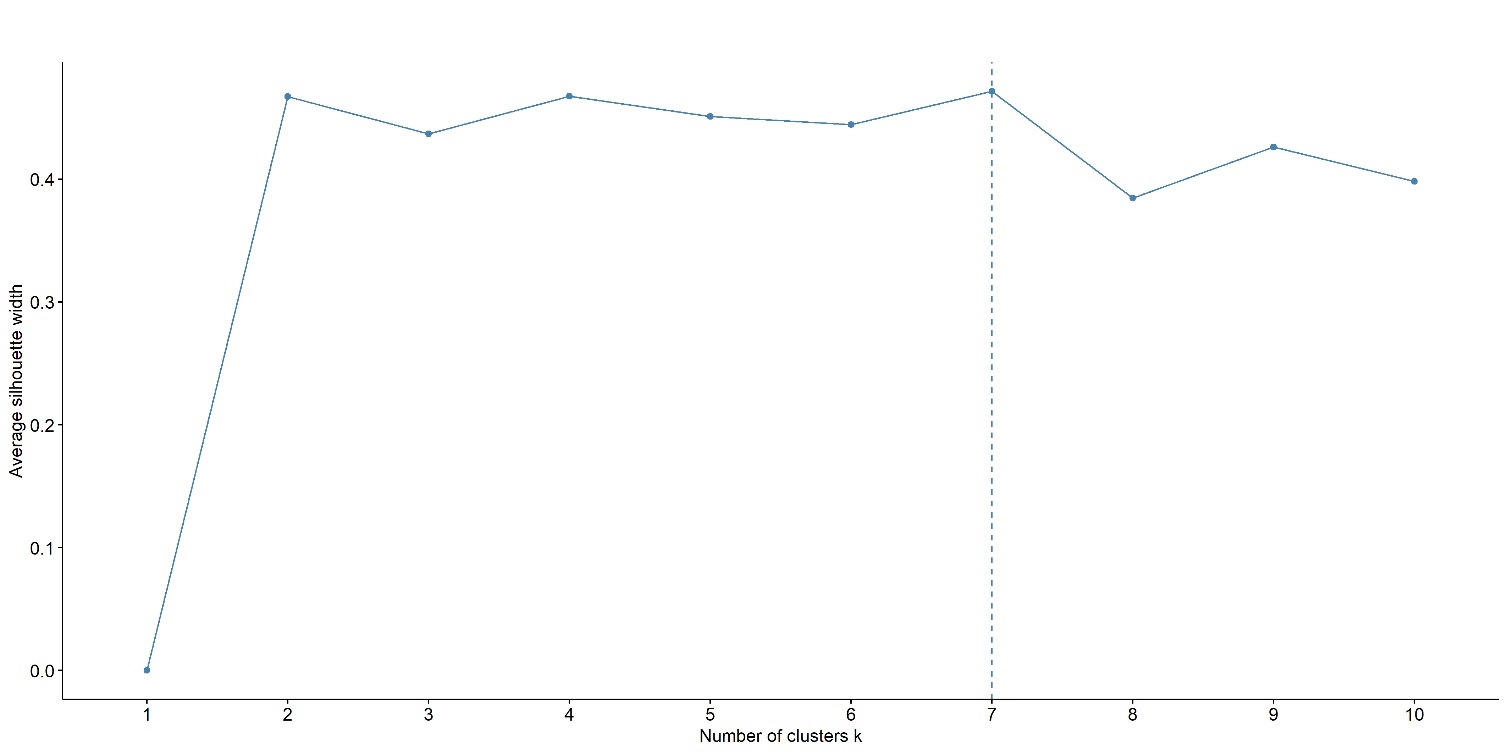


Figure A1. The silhouette method for estimating clustering groups. The plot illustrates the relationship between the average silhouette width on the y-axis and the number of clusters on the x-axis for the investigated data set. The dashed blue line denotes the selected estimated number of clusters used in the k-means algorithm. This figure was made with the R software package called ggplot2, version 3.3.2: Wickham; ggplot2: Elegant Graphics for Data Analysis. Springer-Verlag New York, 2016: <https://ggplot2.tidyverse.org>


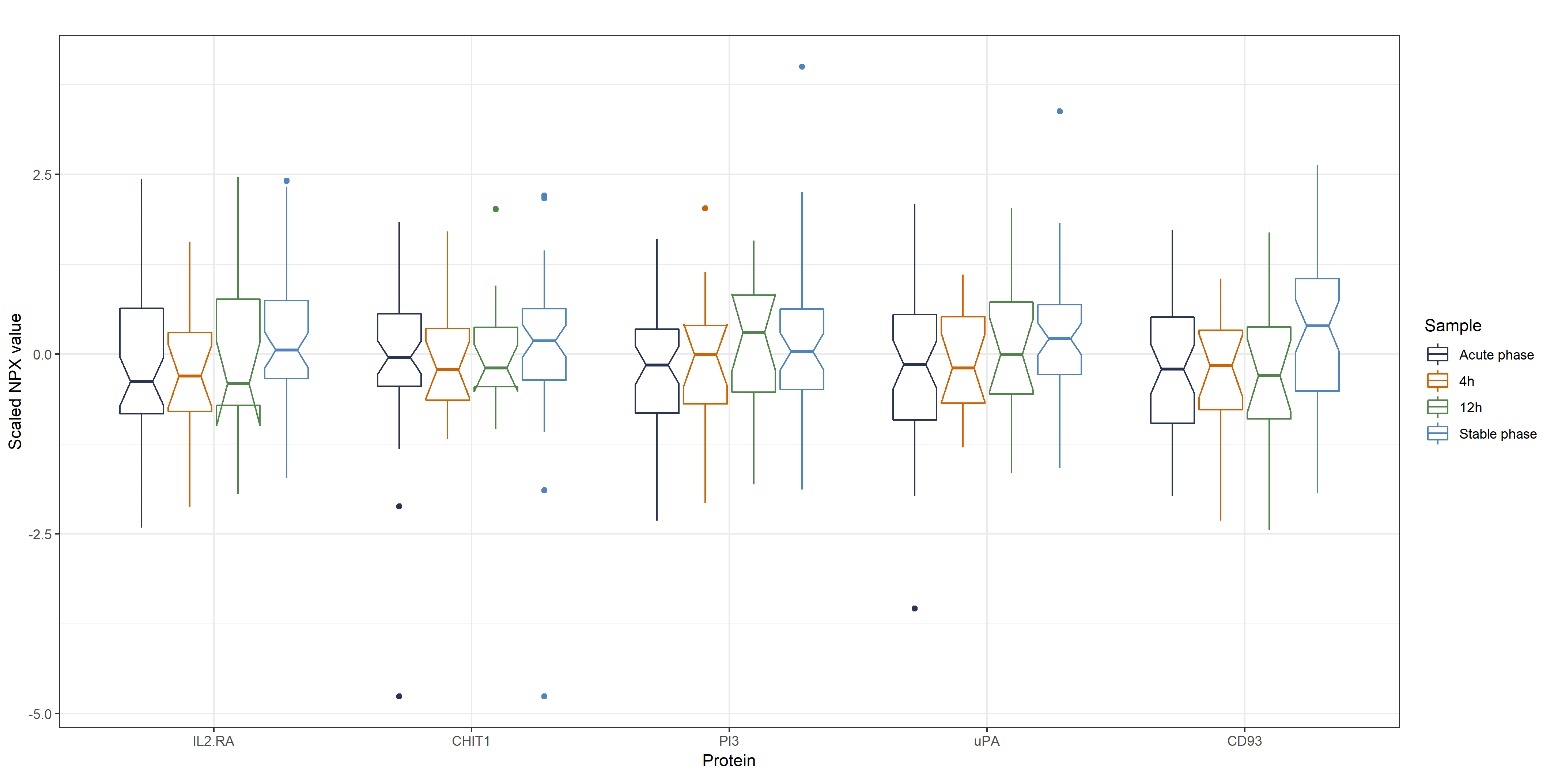


Figure A2. Boxplots for the time points acute phase, 4h, 12h and stable phase for the five proteins in clustering group 2. Notches determines the 95% confidence interval for the median value. On the y-axis scaled NPX values are shown. The scaled NPX values cannot be compared directly between proteins, but only between measurements for the same protein. This figure was made with the R software package called ggplot2, version 3.3.2: Wickham; ggplot2: Elegant Graphics for Data Analysis. Springer-Verlag New York, 2016: <https://ggplot2.tidyverse.org>


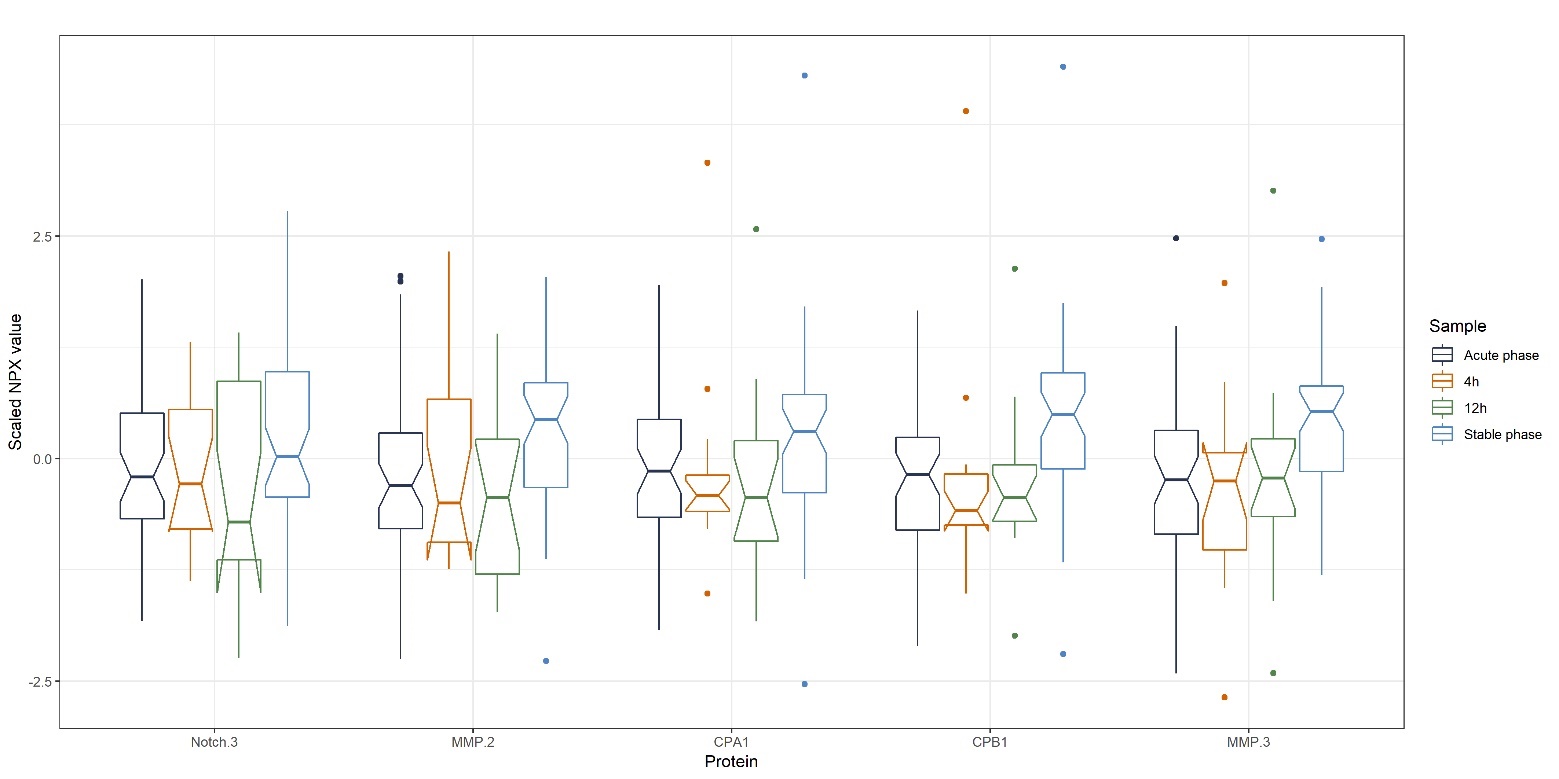


Figure A3. Boxplots for the time points acute phase, 4h, 12h and stable phase for the five proteins in clustering group 2. Notches determines the 95% confidence interval for the median value. On the y-axis scaled NPX values are shown. The scaled NPX values cannot be compared directly between proteins, but only between measurements for the same protein. This figure was made with the R software package called ggplot2, version 3.3.2: Wickham; ggplot2: Elegant Graphics for Data Analysis. Springer-Verlag New York, 2016: <https://ggplot2.tidyverse.org>


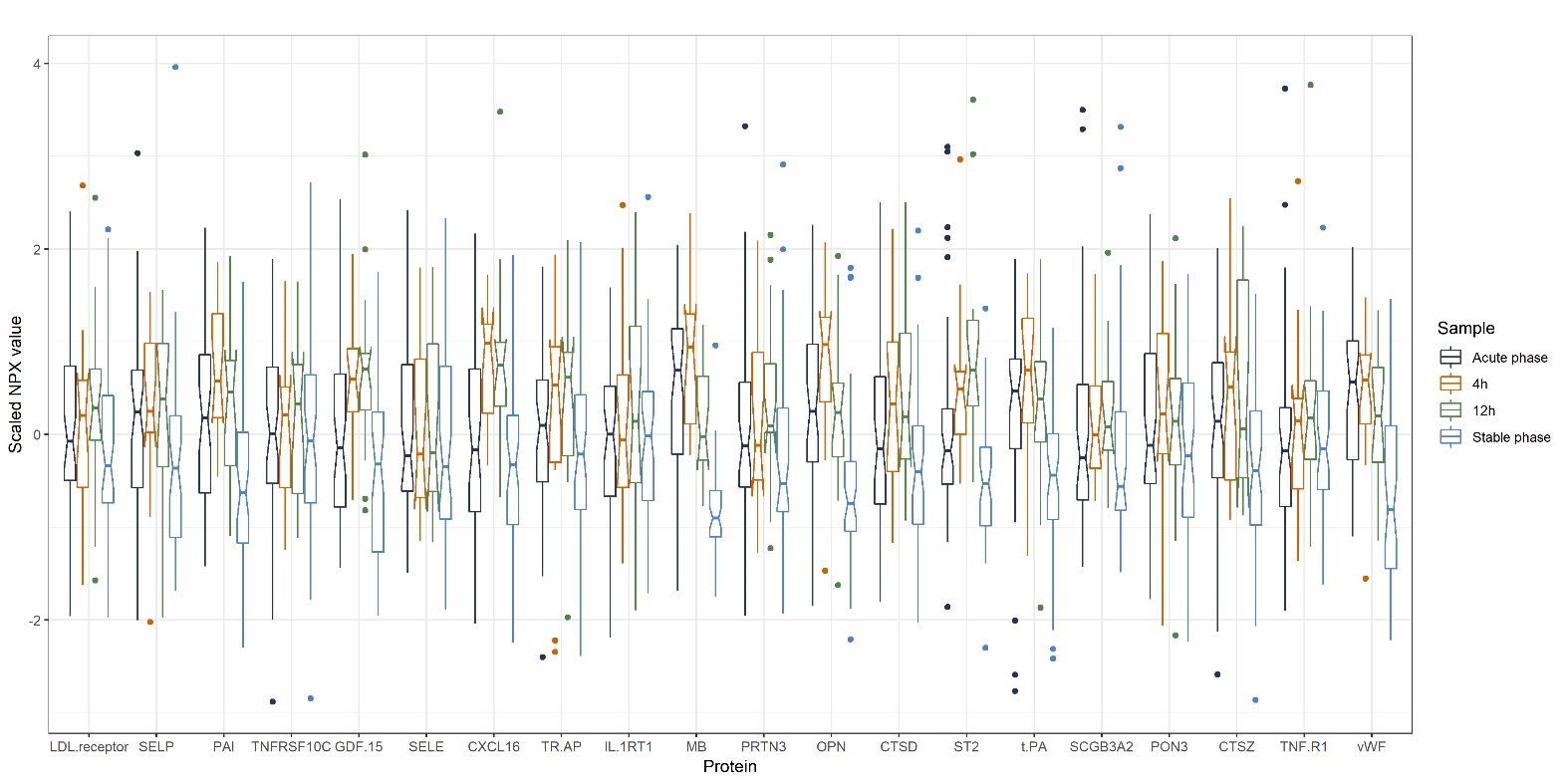


Figure A4. Boxplots for the time points acute phase, 4h, 12h and stable phase for the five proteins in clustering group 2. Notches determines the 95% confidence interval for the median value. On the y-axis scaled NPX values are shown. The scaled NPX values cannot be compared directly between proteins, but only between measurements for the same protein. This figure was made with the R software package called ggplot2, version 3.3.2: Wickham; ggplot2: Elegant Graphics for Data Analysis. Springer-Verlag New York, 2016: <https://ggplot2.tidyverse.org>


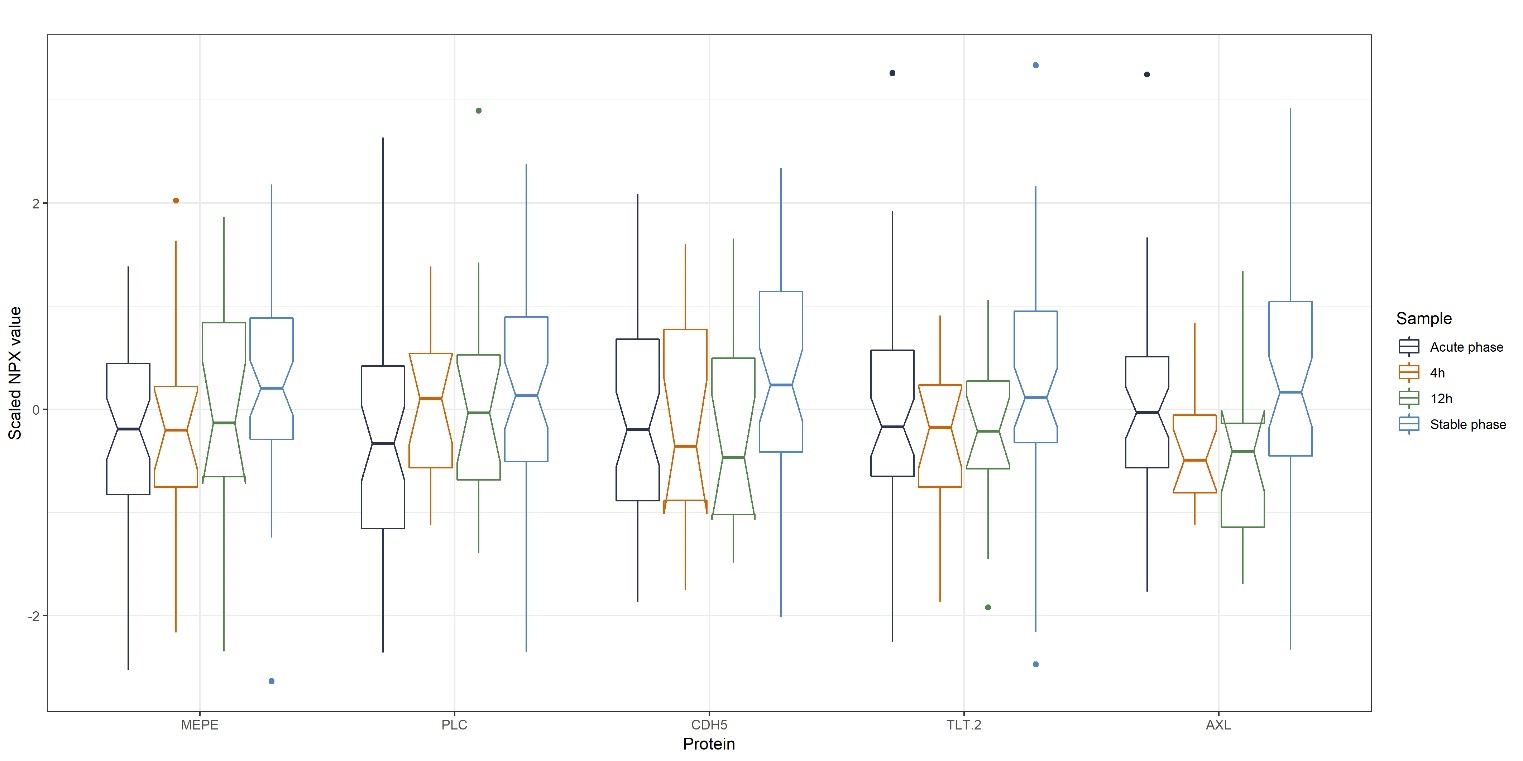


Figure A5. Boxplots for the time points acute phase, 4h, 12h and stable phase for the five proteins in clustering group 2. Notches determines the 95% confidence interval for the median value. On the y-axis scaled NPX values are shown. The scaled NPX values cannot be compared directly between proteins, but only between measurements for the same protein. This figure was made with the R software package called ggplot2, version 3.3.2: Wickham; ggplot2: Elegant Graphics for Data Analysis. Springer-Verlag New York, 2016: <https://ggplot2.tidyverse.org>


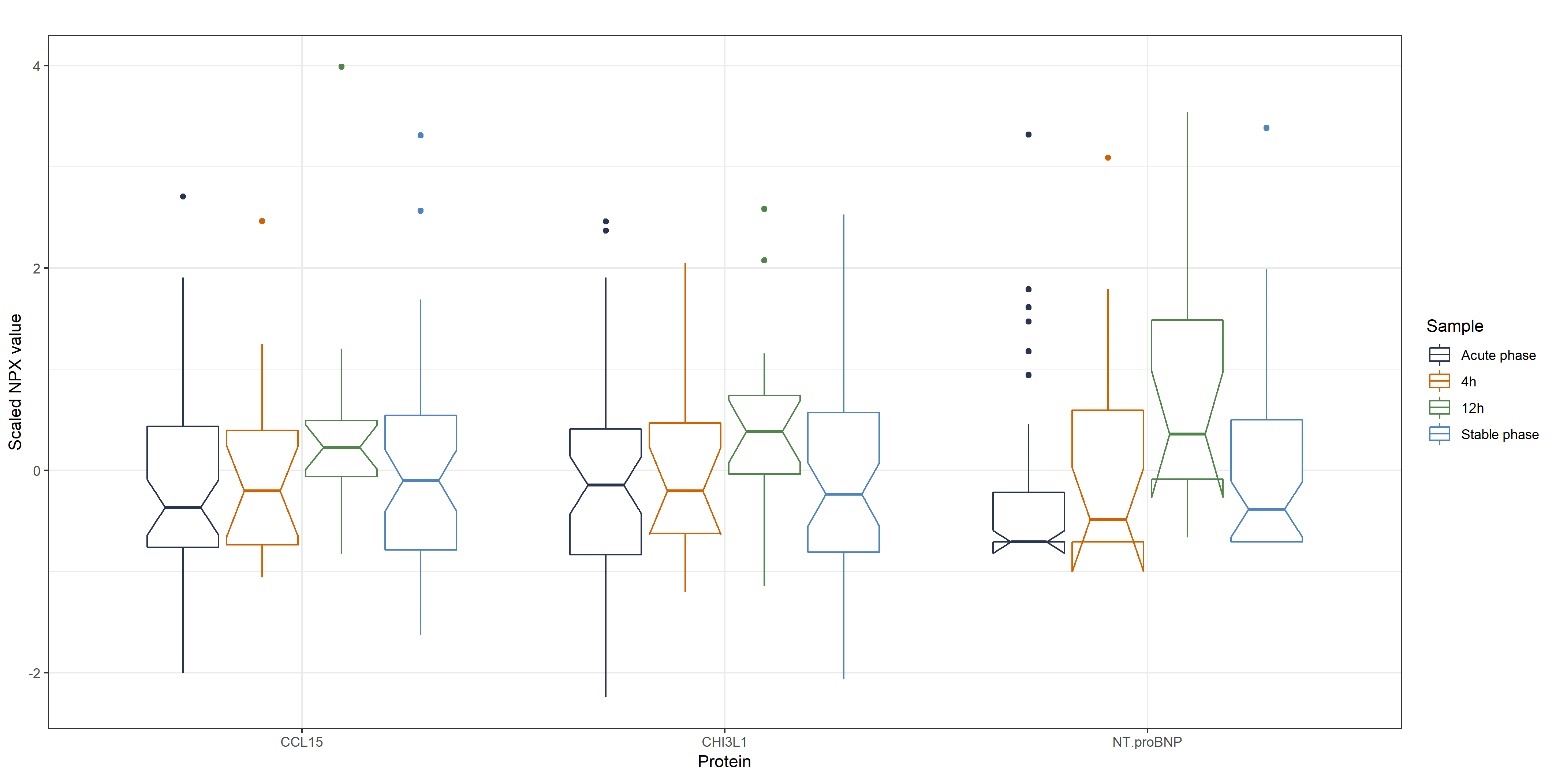


Figure A6. Boxplots for the time points acute phase, 4h, 12h and stable phase for the five proteins in clustering group 2. Notches determines the 95% confidence interval for the median value. On the y-axis scaled NPX values are shown. The scaled NPX values cannot be compared directly between proteins, but only between measurements for the same protein. This figure was made with the R software package called ggplot2, version 3.3.2: Wickham; ggplot2: Elegant Graphics for Data Analysis. Springer-Verlag New York, 2016: <https://ggplot2.tidyverse.org>
